# Performance Evaluation of HDPE-Bakelite Dual-Modified Asphalt Mixtures for Sustainable Pavements

**DOI:** 10.3390/polym17223065

**Published:** 2025-11-19

**Authors:** Muhammad Yasir, Naqeeb Ullah Khattak, Inamullah Khan, Menglim Hoy

**Affiliations:** 1 National Institute of Transportation (NIT), National University of Sciences & Technology (NUST), NUST Campus, Risalpur 24080, Pakistan; muhammadyasir3364@gmail.com (M.Y.); inamullah.khan@nit.nust.edu.pk (I.K.); 2 Department of Civil and Environmental Engineering, South Dakota State University, Brookings, SD 57007, USA; naqeebullah.khattak@jacks.sdstate.edu; 3 School of Civil Engineering, Institute of Engineering, Suranaree University of Technology, Nakhon Ratchasima 30000, Thailand

**Keywords:** HDPE modification, bakelite modifier, asphalt mixture, rutting resistance, moisture susceptibility, resilient modulus, sustainable pavements

## Abstract

Flexible pavements using conventional bitumen are prone to suffering severe distress in hot climates, particularly rutting and moisture-induced damage. This study explores synergistic effects of waste-derived High-Density Polyethylene (HDPE) and Bakelite as dual modifiers for asphalt mixtures under Pakistan’s extreme climate, where summer temperatures exceed 45 °C. Modified mixtures were prepared via wet process using HDPE (3%, 6%, 9% by weight of optimum bitumen content) combined with 6% Bakelite, evaluated against control mixtures (60/70 bitumen, NHA Class-B gradation). Performance assessment included indirect tensile strength, moisture susceptibility (TSR), resilient modulus, and Hamburg wheel tracking tests. The optimal 6%HDPE + 6%Bakelite formulation achieved remarkable improvements over control: 24.7% higher dry ITS (0.647 MPa), 48.7% higher conditioned ITS (0.617 MPa), 95.36% TSR (19% above specifications), 43.7% greater resilient modulus (4866 MPa), and 27.4% lower rutting depth (2.38 mm). These enhancements are likely associated with the development of a stiffer polymer resin network between HDPE and rigid Bakelite particles, which appears to provide a favorable balance between mixture flexibility and stiffness. At 9% HDPE, performance degradation in strength and moisture-related properties suggests possible phase separation, although rutting resistance continued improving. This dual-modification strategy provides sustainable, cost-effective enhancement of pavement durability in hot climates while addressing waste management challenges, offering significant potential for reducing maintenance costs and extending service life.

## 1. Introduction

Roads and highways are essential infrastructures that support national economies by facilitating trade, transportation, and social connectivity. Flexible pavements, which are mainly constructed with asphalt binders, are the most widely used due to their cost-effectiveness and riding comfort [1,2]. However, these pavements face significant challenges from increasing traffic loads and extreme climatic conditions, leading to premature distress, including rutting, fatigue cracking, thermal cracking, and moisture-induced damage [3].

In Pakistan, where summer temperatures regularly exceed 45 °C in southern regions and winter temperatures drop below −5 °C in northern areas, pavement performance challenges are particularly severe [4]. The country’s road network, spanning approximately 263,775 km, experiences substantial stress from both climatic extremes and increasing traffic volumes, with commercial vehicle traffic growing at 8.2% annually [5,6]. The National Highway Authority (NHA) reported maintenance expenditures of PKR 53.48 billion (approximately USD 200 million) in 2022 for maintaining just 12,300 km of national highways, representing a 23% increase from the previous year [7]. This substantial and growing financial burden underscores the urgent need for improved pavement technologies capable of withstanding Pakistan’s challenging service conditions.

The predominant use of conventional penetration-graded bitumen (60/70 and 80/100) in Pakistani highway construction exacerbates these challenges. These binders exhibit significant temperature susceptibility, with viscosity reductions of up to 80% at 60 °C, leading to permanent deformation, while becoming brittle below 10 °C, causing thermal and fatigue cracking [8,9]. Laboratory studies have shown that conventional 60/70 bitumen loses approximately 65% of its complex modulus when the temperature increases from 25 °C to 60 °C, directly correlating with reduced rutting resistance [10]. Consequently, pavements constructed with these materials typically require major rehabilitation within 7–10 years, compared to the design life of 20 years [11].

To address these performance limitations, researchers have explored various modification strategies over the past three decades. These approaches include the implementation of performance-based design systems (Superpave), incorporation of fibers and nanomaterials, chemical modification, and polymer modification [3,12]. Among these strategies, polymer modification has emerged as the most effective for enhancing rheological properties, with studies demonstrating improvements in rutting resistance by 30–50%, fatigue life extension by 2–3 times, and significant enhancement in temperature susceptibility [13,14,15].

Polymer modifiers are categorized into two primary classes: elastomers and plastomers. Elastomers, such as Styrene-Butadiene-Styrene (SBS) and Styrene-Butadiene Rubber (SBR), enhance flexibility and elastic recovery, with SBS-modified binders showing elastic recovery values exceeding 75% compared to less than 10% for unmodified binders [16]. Plastomers, including polyethylene (PE) and polypropylene (PP), improve high-temperature stiffness and deformation resistance, with studies reporting complex modulus increases of 40–60% at 60 °C [17].

Recently, waste-derived polymers have gained significant attention, offering dual benefits of performance enhancement and environmental sustainability. The global generation of plastic waste exceeds 350 million tons annually, with only 9% being recycled [18]. Utilizing these waste materials in road construction provides an effective waste management strategy while improving pavement performance.

High-Density Polyethylene (HDPE), a thermoplastic polymer constituting approximately 12% of global plastic waste, demonstrates excellent potential for asphalt modification [19]. With a melting point range of 120–130 °C and a density of 0.94–0.96 g/cm^3^, HDPE provides thermal stability and forms a three-dimensional polymer network within the asphalt matrix [20]. When incorporated at optimal concentrations, HDPE has been shown to increase Marshall stability and reduce rutting [21].

Mansourian et al. [22] investigated HDPE nanocomposites in asphalt binders, reporting a 45% improvement in rutting parameter (G */sin δ) at 64 °C while maintaining adequate low-temperature performance. Similarly, Ullah et al. [23] found that HDPE modification at 5% by weight of bitumen enhanced the resilient modulus by 38% and reduced moisture susceptibility by 18%. The mechanism of HDPE modification involves the formation of a continuous polymer phase at concentrations above 4%, creating a polymer-rich network that resists deformation [22].

Bakelite powder, a thermosetting phenol-formaldehyde resin obtained from industrial waste, offers complementary properties for asphalt modification. With a decomposition temperature of 270–350 °C and specific gravity of 1.36, Bakelite acts as both a filler and modifier, enhancing mixture stiffness through its cross-linked molecular structure [24]. Unlike thermoplastics, Bakelite cannot be remelted once cured, but its incorporation as a fine powder passing the 150 µm (#100 sieve) ensures uniform distribution within the asphalt matrix [25].

Recent studies on Bakelite modification have shown promising results. Ali et al. [3] reported that 6% Bakelite addition increased Marshall stability by 22% and improved the tensile strength ratio from 80% to 88%, indicating enhanced moisture resistance. Ahmad et al. [24] observed that combining Bakelite with crumb rubber nearly doubled Marshall stability compared to control mixtures. The enhancement mechanism involves Bakelite particles acting as stress transfer points, improving aggregate–binder adhesion through mechanical interlocking [26].

Despite individual successes of HDPE and Bakelite modifications, both materials exhibit limitations when used alone. Excessive HDPE content (>7%) leads to phase separation, storage instability, and poor workability at conventional mixing temperatures [27]. Conversely, while Bakelite increases stiffness and moisture resistance, it reduces mixture flexibility and may increase brittleness at low temperatures [3].

Critically, the literature reveals a significant gap: no comprehensive studies have investigated the synergistic effects of combining HDPE and Bakelite as dual modifiers. The potential complementary nature of these materials, flexibility from HDPE and rigidity from Bakelite, suggests that their combination could yield superior performance compared to single modifications. Furthermore, limited research has simultaneously evaluated multiple performance parameters, including resilient modulus, indirect tensile strength, moisture susceptibility, and rutting resistance for such hybrid modifications under conditions relevant to hot climates.

This research aims to design and evaluate asphalt mixtures modified with high-density polyethylene (HDPE) and Bakelite for enhanced performance in hot climate conditions. The study involves determining the optimum bitumen content (OBC) using the Marshall mix design method, preparing both control and modified specimens with varying HDPE–Bakelite proportions, and conducting key laboratory tests including Resilient Modulus (MR), Indirect Tensile Strength (ITS), Hamburg Wheel Tracking Test (rutting resistance), and Moisture Susceptibility (TSR). The performance outcomes of the modified mixtures will then be compared with the control mix to identify the most effective HDPE–Bakelite combination for sustainable pavement applications.

The findings of this research have practical implications for highway agencies and engineers seeking cost-effective solutions to extend the lifespan of pavements under severe traffic and climatic conditions.

## 2. Materials and Methods

### 2.1. Materials

Aggregates for this study were obtained from Babuzai quarry, Mardan District of Khyber Pakhtunkhwa, Pakistan. The aggregates were selected following the hot mix asphalt pavement standard specifications. Both coarse and fine aggregates underwent laboratory characterization according to ASTM and AASHTO standards to ensure compliance with NHA specifications. Table 1 presents the physical properties of the aggregates used in this study. The study utilized 60/70 penetration grade bitumen, the most widely used grade in Pakistan, sourced from Attock Oil Refineries, Rawalpindi. The bitumen was tested for compliance with standard specifications as shown in Table 2.

HDPE was obtained in granular form from a local cooler factory in Peshawar, Pakistan. The material was used as received without further processing. The physical properties of HDPE, including density, softening point, tensile strength at yield, and flexural modulus, were taken from the manufacturer’s technical datasheet and are summarized in Table 3. Bakelite (phenol-formaldehyde resin) was procured in ground form from Azmat Polymers PVT Ltd., Gujranwala, Pakistan. The material was sieved, and the portion passing through sieve #100 (150 μm) was used for modification. Table 4 shows the properties of Bakelite powder.

### 2.2. Mix Design and Sample Preparation

A comprehensive experimental methodology was adopted in this research, utilizing a systematic parallel framework for developing both control and HDPE-Bakelite dual-modified asphalt mixtures.

The research methodology commenced with simultaneous characterization of materials, where aggregates underwent testing for physical properties (specific gravity, water absorption), shape characteristics (elongation and flakiness indices), and strength parameters (aggregate impact value and Los Angeles abrasion), while the base bitumen was evaluated for penetration, ductility, softening point, specific gravity, and flash/fire points according to ASTM and AASHTO standards. Following material characterization, the methodology diverged into two parallel paths for control and modified mixture development.

For the control mixture, aggregates meeting specifications were batched according to NHA Class-B gradation (Table 5) for wearing course applications, and ARL 60/70 grade bitumen was added at varying contents (4.0%, 4.5%, 5.0%, 5.5%, and 6.0% by weight of aggregate). The Marshall mix design method was employed to determine the optimum bitumen content (OBC) based on maximum stability, target air voids of 4%, maximum unit weight, and flow values within the 2–4 mm specification range. The modified mixture development followed an identical aggregate preparation process to ensure direct comparability; however, the base bitumen underwent modification through the wet process before incorporation. The modification protocol involved heating the bitumen to 150–160 °C, gradually adding HDPE granules at three levels (3%, 6%, and 9% by weight of OBC) with continuous mixing at 500 rpm for 30 min at 145 °C, followed by Bakelite powder addition (maintained constant at 6% by weight of OBC) and continued mixing at 400 rpm for 15 min, with the temperature maintained at 155–165 °C throughout the process.

### 2.3. Performance Experimental Program

#### 2.3.1. Indirect Tensile Strength Test

The indirect tensile strength (ITS) test was conducted in accordance with ASTM D6931 [37]. The specimens were prepared using Marshall compaction (75 blows per face) to achieve target dimensions of 100 mm diameter and 65 mm height. The specimens were stored at room temperature, then immersed in a water bath at 25 ± 0.5 °C for 2 h before ITS testing. The specimens were loaded along the vertical diametral plane at a constant deformation rate of 50 mm/min until failure. The maximum load was recorded and converted to tensile strength using:

(1) $$ITS=\frac{2P}{\pi dt},$$

where *P* = maximum load at failure (N), *d* = specimen diameter (mm), and *t* = specimen thickness (mm). Three replicates were tested per mixture, and the ITS values were used as a baseline for tensile cracking resistance.

The dry (unconditioned) ITS values served as a reference for calculating the tensile strength ratio and evaluating the strengthening effect of polymer modification. The wet (conditioned) ITS testing assessed the mixture’s retained strength after moisture exposure, simulating long-term field conditions. Three additional specimens per mixture were subjected to moisture conditioning following the AASHTO T283 [38] protocol. Subjected to partial vacuum saturation (70–80% degree of saturation), wrapped, and frozen at −18 ± 3 °C for 16 h, followed by immersion in a 60 ± 1 °C water bath for 24 h [28]. After conditioning, specimens were stabilized in a 25 °C water bath for 2 h before ITS testing. Moisture resistance was quantified through the tensile strength ratio (TSR), calculated as:

(2) $$TSR=\frac{{ITS}_{conditioned}}{{ITS}_{unconditioned}}\times 100,$$

#### 2.3.2. Resilient Modulus Test

The resilient modulus (M_R_) of asphalt mixtures was determined according to ASTM D4123 [39], which evaluates the elastic and viscoelastic response of bituminous mixes under repeated indirect tensile loading. Although asphalt mixtures exhibit both elastic and plastic behavior, the test assumes that under small repeated loads, most of the deformation is recoverable and can therefore be considered elastic.

In this test, cylindrical specimens were subjected to repeated haversine loading at a frequency of 2 Hz, consisting of 0.1 s of loading followed by a 0.4-s recovery period. Based on laboratory observations, 100 load repetitions were applied to each specimen, ensuring that resilient deformations reached a stable condition.

The horizontal recoverable deformation was measured using two Linear Variable Differential Transformers (LVDTs) mounted at mid-height, positioned diametrically opposite on the specimen’s surface. The resilient modulus was then calculated using the following equation:

(3) $$M_{R}=\frac{P(\mu +0.27)}{t\delta_{t}},$$

where, *P* = maximum dynamic load (N), μ = Poisson’s ratio (assumed as 0.35), *t* = specimen thickness (mm), $\delta_{t}\;$ = total recoverable horizontal deformation (mm).

Three replicate specimens were tested for each mixture type. Higher Mr values indicate improved stiffness and load-distribution capacity, while excessively high stiffness may suggest increased brittleness.

#### 2.3.3. Hamburg Wheel Tracking Test

Rutting resistance evaluation employed the Hamburg Wheel Tracking Test (AASHTO T324 [40])to assess permanent deformation susceptibility under the combined effects of loading, temperature, and moisture. Two cylindrical specimens per mixture were prepared using Superpave Gyratory Compaction to achieve 150 mm diameter, 62.5 mm height, and 7% ± 0.5% air voids, representing typical field density conditions.

Specimens underwent conditioning in a 40 ± 1 °C water bath for 30 min before testing, ensuring complete thermal equilibration. The test configuration positioned specimens in steel molds submerged in 40 °C water throughout testing, simulating worst-case field conditions. A steel wheel (203.2 mm diameter, 47 mm width) applied a 705 ± 22 N load while traversing the specimen surface at 52 ± 2 passes per minute, with one pass defined as forward and backward movement across the specimen.

Rut depth measurements were recorded continuously using displacement transducers with 0.01 mm resolution, capturing data at 500-pass intervals through 5000 total passes. The test generated three key parameters: (i) maximum rut depth at 5000 passes, indicating overall rutting resistance; (ii) stripping inflection point (if observed), marking moisture damage initiation; and (iii) creep slope, representing deformation rate in the linear portion of the curve. Test termination occurred at 5000 passes or upon reaching 12.5 mm rut depth, whichever occurred first. Lower rut depths and creep slopes indicated superior rutting resistance, with the 12.5 mm threshold representing a failure criterion for highway applications.

## 3. Results and Discussion

### 3.1. Indirect Tensile Strength (ITS) Results

**Figure 1:**
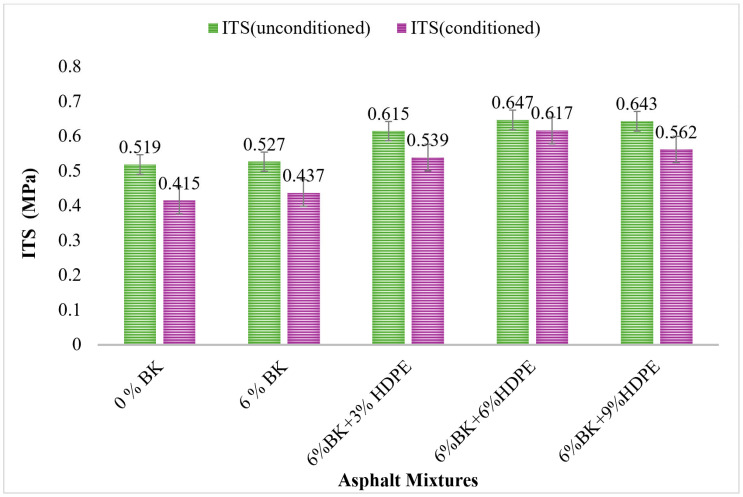
ITS results of modified asphalt mixtures.

Figure 1 presents the unconditioned and moisture-conditioned indirect tensile strength (ITS) values for control and modified asphalt mixtures. The incorporation of BK and HDPE modifiers significantly enhanced ITS performance under both dry and wet conditions, with unconditioned values consistently exceeding conditioned values due to moisture-induced damage during AASHTO T283 [38] conditioning.

Addition of 6% BK alone resulted in modest improvements (0.527 MPa unconditioned, 0.437 MPa conditioned), representing only 1.5% and 5.3% gains over control. The moisture-induced strength loss decreased to 17.1%. As a thermosetting phenol-formaldehyde resin, Bakelite provides limited enhancement through three mechanisms: (1) rigid filler densification, reducing deformable binder volume, (2) mechanical interlocking at aggregate–binder interfaces via irregular particle morphology, and (3) stiffness increases from its high flexural modulus (~8000 kg/cm^2^). However, Bakelite’s cross-linked structure prevents molecular-level integration with bitumen, limiting reinforcement efficiency to mechanical contact rather than polymer chain interpenetration [3,24].

The 6%BK + 3%HDPE combination achieved substantial improvements (0.615 MPa unconditioned, 0.539 MPa conditioned), representing 18.5% and 29.9% gains over control. This far exceeds the additive contributions of BK alone (1.5%), demonstrating clear synergistic effects.

The optimal performance was achieved with 6%BK + 6%HDPE: 0.647 MPa (unconditioned) and 0.617 MPa (conditioned), representing remarkable 24.7% and 48.7% improvements over control. At 6% HDPE concentration, the polymer exceeds the critical percolation threshold (4–5% by weight), forming a continuous three-dimensional network throughout the asphalt matrix. This creates optimal synergy where: (1) the continuous HDPE network provides a reinforced skeleton resisting deformation, (2) rigid Bakelite particles become embedded within the polymer matrix as effective reinforcing nodes, and (3) extensive molecular entanglements develop between HDPE chains (MW: 50,000–250,000 g/mol) and around Bakelite particles, creating physical crosslinks that dramatically increase tensile failure resistance [27].

The 6%BK + 9%HDPE mixture showed decreased performance, suggesting excessive HDPE content leads to phase separation and storage instability. At concentrations above 7%, HDPE-modified bitumen exhibits dramatic increases in separation indices, creating excessively stiff polymer-rich phases and weak polymer-depleted zones where failure preferentially occurs during testing [41].

**Figure 2:**
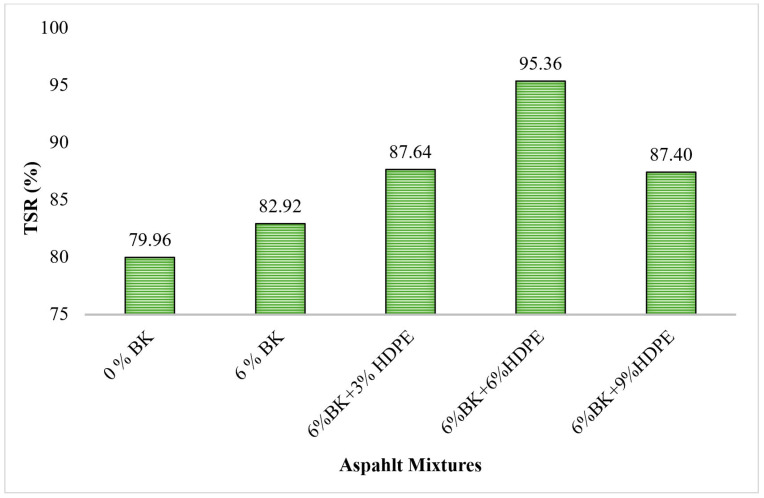
Tensile strength ratio (TSR) of modified asphalt mixtures.

Figure 2 presents the tensile strength ratio (TSR) values for control and modified asphalt mixtures, which quantifies the moisture damage susceptibility by measuring the retained strength after moisture conditioning. According to AASHTO T283 specifications, a minimum TSR value of 80% is required for adequate moisture resistance in asphalt pavements.

The optimal moisture resistance was achieved with the 6%BK + 6%HDPE combination, which exhibited an exceptional TSR value of 95.36%. This represents a remarkable 19.2% improvement over the control mixture and 15.0% improvement over BK-only modification, far exceeding the minimum AASHTO requirement. Khan et al. [42] achieved TSR improvement from 79.53% to 97.14% with 4.5%SBS + 6%Nanoclay, while Khattak et al. [30] reported TSR improvements with 6%BK + 4%Nanoclay modification. The 6%BK + 6%HDPE combination in the current study demonstrates comparable performance to these advanced modification systems while offering the advantage of utilizing waste-derived HDPE.

However, the mixture containing 6%BK + 9%HDPE showed a decrease in TSR to 87.40%, still well above the control but significantly lower than the optimal 6%BK + 6%HDPE combination. This 8.3% reduction suggests that excessive HDPE content compromises moisture resistance, likely due to several factors: (1) potential phase separation at high polymer concentrations, (2) reduced aggregate–binder interfacial bonding due to excessive polymer coating, and (3) possible agglomeration effects that create weak points in the mixture structure. This trend aligns with the established understanding that HDPE content above 7% can lead to storage instability and performance degradation.

### 3.2. Resilient Modulus Results

**Figure 3:**
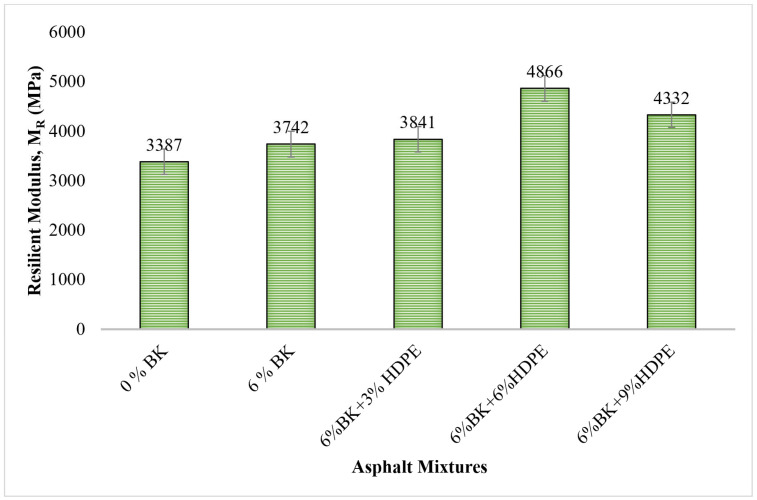
Resilient modulus of asphalt mixtures.

Figure 3 presents the resilient modulus values for control and modified asphalt mixtures, representing the elastic stiffness and load-distribution capacity under repeated loading conditions. The resilient modulus is a critical parameter for pavement design, directly influencing stress distribution within pavement layers and predicting long-term structural performance under cyclic traffic loading.

This finding aligns with previous research by Khattak et al. [43], who reported that 6%BK + 4%Nanoclay modification improved resilient modulus by 60% (from 3387 MPa to 5413 MPa). The substantially greater improvement achieved with nanoclay addition (compared to BK alone) demonstrates that nanoscale reinforcing agents with high aspect ratios provide superior stiffness enhancement compared to microscale rigid fillers, primarily through their ability to create extensive interfacial regions and tortuous load transfer pathways.

The transition from 6%BK (3742 MPa) to 6%BK + 3%HDPE (3841 MPa) shows only modest additional improvement (2.6%), indicating that 3% HDPE is insufficient to fundamentally alter the mixture’s elastic stiffness characteristics. At this concentration, the performance trends suggest that HDPE may form isolated polymer-rich domains with incipient network connections, although the mixture likely does not yet reach the critical percolation threshold required for a fully continuous network. The resilient modulus enhancement at 3% HDPE derives primarily from localized stiffening where polymer domains exist, but the overall response remains dominated by the continuous bitumen-Bakelite matrix.

The dramatic increase from 6%BK + 3%HDPE (3841 MPa) to 6%BK + 6%HDPE (4866 MPa) represents a 26.7% jump and signifies a critical microstructural transition. This substantial enhancement corresponds to the achievement of a continuous three-dimensional HDPE network that fundamentally transforms the load-bearing mechanisms of the composite system. The exceptional resilient modulus of 4866 MPa at 6%BK + 6%HDPE (43.7% improvement over control) reflects three synergistic reinforcement mechanisms: At 6% HDPE, the polymer concentration exceeds the percolation threshold, creating a continuous three-dimensional network that provides a distinct load-bearing pathway with substantially higher elastic modulus than the bitumen phase. The 1:1 ratio of Bakelite to HDPE creates an optimally balanced dual-phase reinforcement system. Bakelite particles serve as rigid nodes embedded within the HDPE network, analogous to crosslink points in chemically crosslinked elastomers. This hierarchical resistance mechanism—combining HDPE network elastic deformation, Bakelite particle constraint of polymer chain mobility, and particle-to-particle stress transfer—is more efficient than either modifier alone. The extensive polymer network at 6% HDPE restricts molecular mobility and viscous flow processes, resulting in more elastic (less viscous) behavior. At the test frequency (2 Hz) and temperature (25 °C), the HDPE-modified system exhibits solid-like behavior with a higher storage modulus and a lower phase angle compared to unmodified bitumen.

However, the 6%BK + 9%HDPE mixture exhibited M_R_ = 4332 MPa, representing an 11.0% decrease from the optimal composition. At 9% HDPE, polymer-bitumen incompatibility leads to macroscopic phase separation, creating polymer-rich zones and polymer-depleted zones. The polymer-depleted zones exhibit lower stiffness, increasing measured horizontal deformation and reducing calculated resilient modulus. Attaelmanan et al. [41] demonstrated that HDPE concentrations exceeding 7% exhibit poor storage stability with severe phase segregation. Furthermore, High viscosity at 9% HDPE creates workability challenges, making it difficult to achieve uniform aggregate coating and proper densification during Marshall compaction. Inadequate compaction results in higher air voids and reduced load transfer efficiency. Excessive polymer network density causes embrittlement, where the material develops microcracks under repeated loading rather than responding elastically. The overly stiff structure accumulates microstructural damage during the 100 loading cycles, increasing deformation and reducing resilient modulus [44].

### 3.3. Hamburg Wheel Tracking Test (HWTT)—Rutting Resistance

**Figure 4:**
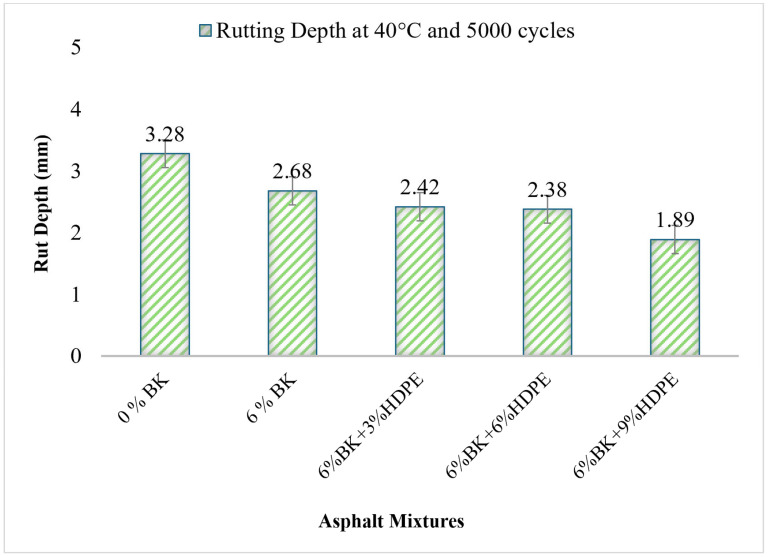
Rut depth of asphalt mixtures.

Figure 4 presents the rutting depth measurements for control and modified asphalt mixtures after 5000-wheel passes at 40 °C, representing permanent deformation resistance under simulated traffic loading conditions. Rutting depth is inversely related to performance, which indicates that lower values indicate superior resistance to permanent deformation, a critical distress mode in hot climate pavements like those in Pakistan, where summer temperatures regularly exceed 45 °C.

The control mixture (0% BK) exhibited a rutting depth of 3.28 mm, indicating moderate susceptibility to permanent deformation under the test conditions. This baseline performance is typical of conventional 60/70 penetration grade bitumen, which exhibits significant viscosity reduction and stiffness loss at elevated temperatures. At 40 °C, conventional bitumen becomes sufficiently soft so that the asphalt mixture cannot adequately resist the shear stresses induced by repeated wheel loading, resulting in progressive accumulation of permanent deformation.

The addition of 6% BK alone reduced rutting depth to 2.68 mm, representing an 18.3% improvement over the control mixture. This enhancement confirms Bakelite’s effectiveness in improving high-temperature stability through two mechanisms: (1) increased mixture stiffness via rigid particle reinforcement, and (2) enhanced aggregate–binder adhesion through mechanical interlocking. The improvement aligns with Yousaf et al. [3], who reported that 6% Bakelite enhanced rut resistance by 29% and 38% for 12 mm and 19 mm NMAS mixtures, respectively. However, the modest improvement indicates that rigid filler modification alone provides limited rutting resistance enhancement compared to polymer network-based modifications.

Contrary to the ITS and resilient modulus results, where 6% HDPE was optimal, the rutting resistance continues to improve at 9% HDPE, achieving the best performance (1.89 mm rut depth). This divergent trend reveals important mechanistic insights about the different failure modes governing tensile strength versus permanent deformation resistance.

At the HWTT test temperature (40 °C), HDPE exists well below its melting point (120–130 °C) and maintains substantial crystallinity and stiffness. The higher polymer content at 9% HDPE provides greater resistance to viscous flow and permanent deformation at this elevated temperature. While 9% HDPE showed embrittlement effects in the ITS test (conducted at 25 °C under rapid tensile loading), the HWTT conditions (40 °C, slow repeated compressive/shear loading) favor the high stiffness provided by excessive polymer content.

Rutting is a viscoplastic failure mode dominated by time-dependent flow under sustained loading, while tensile cracking is a brittle/ductile failure mode governed by cohesive and adhesive strength. The 9% HDPE mixture’s high stiffness effectively resists viscous flow during the 0.1 s wheel loading cycles, even if this same excessive stiffness creates brittleness problems under rapid tensile loading. Research by Moghadas Nejad et al. [45] similarly found that HDPE concentrations optimal for rutting resistance (6–8%) were higher than those optimal for fatigue resistance (4–6%), reflecting these different failure mechanisms.

The divergent optimal compositions for different performance parameters present a practical challenge. For practical implementation, the 6%BK + 6%HDPE composition is recommended as the overall optimal formulation because: (1) It provides excellent rutting resistance (2.38 mm), only 20.6% higher than the best performance, but still 27.4% better than control. (2) It achieves optimal tensile strength, moisture resistance, and resilient modulus. (3) It avoids the phase separation, workability, and brittleness issues associated with 9% HDPE. (4) The marginal rutting improvement from 6% to 9% HDPE (2.38 mm to 1.89 mm, a 0.49 mm difference) does not justify the compromises in other critical properties.

This balanced approach prioritizes overall pavement durability rather than optimizing for a single distress mode, consistent with mechanistic-empirical pavement design principles that consider multiple failure mechanisms simultaneously. These mechanistic interpretations are consistent with recent multiscale investigations showing that additives can tune bitumen structure and performance across scales and align with current sustainability-oriented approaches employing municipal solid-waste–derived polymers, lignin, and antioxidant systems to improve pavement resilience [46].

From a practical standpoint, the selection of an optimal modifier dosage should consider not only performance but also economic feasibility. In this study, Bakelite and HDPE are both derived from waste streams and are used at relatively low dosages with respect to the total mixture, implying a modest increase in initial material cost. When this limited cost increase is viewed alongside the substantial improvements in TSR, resilient modulus, and rutting resistance achieved by the 6% BK + 6% HDPE mixture, it is expected that the dual-modified mixture would provide favorable life-cycle economics through extended service life and reduced maintenance needs. Nevertheless, a detailed performance–cost optimization and life-cycle cost analysis, including local material prices and agency/user costs, is recommended for future studies to support large-scale implementation of HDPE–Bakelite modified asphalt mixtures.

## 4. Conclusions

This study investigated the synergistic effects of waste-derived High-Density Polyethylene (HDPE) and Bakelite powder as dual modifiers for asphalt mixtures intended for hot climate applications. Through comprehensive laboratory evaluation, including indirect tensile strength, moisture susceptibility, resilient modulus, and Hamburg wheel tracking tests, the following conclusions were drawn:

1. The 6%BK + 6%HDPE combination emerged as the optimal formulation, demonstrating balanced improvements across all performance parameters. This composition achieved 24.7% improvement in unconditioned ITS, 48.7% improvement in conditioned ITS, 95.36% TSR (exceeding AASHTO T283 requirements by 19%), 43.7% enhancement in resilient modulus, and 27.4% reduction in rutting depth compared to control mixtures.
2. The superior performance of 6%BK + 6%HDPE results from complementary reinforcement mechanisms. At 6% concentration, HDPE forms a continuous three-dimensional polymer network that provides elastic recovery and flexibility, while Bakelite particles act as rigid reinforcing nodes embedded within this network, enhancing stiffness and load transfer efficiency. This dual-phase system achieves an optimal balance between flexibility and rigidity that neither modifier provides individually.
3. Polymer modification dramatically improved moisture damage resistance, with the optimal 6%BK + 6%HDPE composition achieving a TSR of 95.36% compared to 79.96% for control mixtures. This represents a critical advancement for Pakistani pavements subjected to monsoon conditions and temperature extremes, potentially extending service life by reducing moisture-induced distress, including stripping and raveling.
4. The modified mixtures demonstrated superior performance at elevated temperatures (40 °C HWTT) while maintaining adequate low-temperature properties. The 6%BK + 6%HDPE formulation reduced rutting depth by 27.4%, addressing the primary distress mechanism in Pakistan’s hot climate regions where summer temperatures regularly exceed 45 °C.
5. Increasing HDPE content beyond 6% (i.e., 9%HDPE combinations) resulted in performance degradation in tensile strength and resilient modulus, which may be related to mixture stiffness, embrittlement, and possible phase separation or storage instability of the polymer-modified binder. In contrast, rutting resistance continued improving at 9% HDPE, indicating that the optimum HDPE content can differ depending on the governing distress mechanism or failure mode. Future work will incorporate microstructural analyses to directly observe the internal morphology of HDPE–Bakelite modified binders and validate these proposed mechanisms.

The findings demonstrate that a strategic combination of waste-derived HDPE and Bakelite provides a sustainable, cost-effective approach to enhance asphalt pavement performance in hot climates, offering dual benefits of improved engineering properties and environmental waste management.

While this study demonstrates significant improvements in high-temperature stability, moisture resistance, and load-bearing capacity, direct evaluation of cracking resistance (low-temperature fracture and fatigue testing) was not performed. Given that both HDPE and Bakelite increase mixture stiffness, comprehensive cracking characterization through Semi-Circular Bending tests, fatigue testing, and binder-level low-temperature evaluation is essential for complete performance validation and represents a critical priority for future research.

## Figures and Tables

**Table 1:** Physical properties of aggregates.

Test	Standard	Aggregate		Specification
		Coarse	Fine	
Specific Gravity	ASTM C127/C128 [28]	2.63	2.64	-
Water Absorption (%)	ASTM C127/C128 [28]	0.93	2.61	<3%
Los Angeles Abrasion (%)	ASTM C131 [29]	27	-	<30%
Aggregate Crushing Value (%)	BS 812-110 [30]	19	-	<30%
Flakiness Index (%)	BS 812-105 [31]	8.5	-	<10%
Elongation Index (%)	BS 812-106 [32]	3.85	-	<10%

**Table 2:** Properties of 60/70 Penetration Grade Bitumen.

Test	Standard	Results	Specification
Penetration 25 (°C), mm	ASTM D 5 [33]	63	60–70
Flash point (°C)	ASTM D92 [34]	260	232 (min)
Fire Point (°C)	ASTM D92 [34]	292	270 (min)
Specific gravity	ASTM 70 [35]	1.04	1.01–1.06
Ductility Test, cm	ASTM D113 [36]	123	>100

**Table 3:** Properties of HDPE.

Properties	Results
Density (kg/cm^2^)	0.948–0.953
Softening point (°C)	122
Tensile strength at yield (kg/cm^2^)	190
Flexural modulus (kg/cm^2^)	8000

**Table 4:** Properties of Bakelite.

Properties	Results
Specific gravity	1.36
Melting point range (°C)	150–165
Decomposition temp. range (°C)	270–350
Sieve analysis	Passing sieve#100 (150 µm)

**Table 5:** NHA Class-B aggregate gradation for wearing course mixtures.

Sieve Size	Passing (%)
19 mm (3/4”)	100
12.5 mm (1/2”)	75–90
9.5 mm (3/8”)	60–80
4.75 mm (No. 4)	40–60
2.36 mm (No. 8)	20–40
0.300 mm (No. 50)	5–15
0.075 mm (No. 200)	3–8

## Data Availability

The original contributions presented in this study are included in the article. Further inquiries can be directed to the corresponding author.

## Abbreviations

The following abbreviations are used in this manuscript:

AASHTO	American Association of State Highway and Transportation Officials
ASTM	American Society for Testing and Materials
BK	Bakelite
HDPE	High-Density Polyethylene
HWTT	Hamburg Wheel Tracking Test
ITS	Indirect Tensile Strength
LVDT	Linear Variable Differential Transformer
MR	Resilient Modulus
NHA	National Highway Authority
OBC	Optimum Bitumen Content
PE	Polyethylene
PP	Polypropylene
SBR	Styrene-Butadiene Rubber
SBS	Styrene-Butadiene-Styrene
TSR	Tensile Strength Ratio
